# Autonomic Nervous System Monitoring: Cardiac Magnetic Resonance Imaging Data as a Surrogate for Autonomic Data in Children

**DOI:** 10.7759/cureus.32014

**Published:** 2022-11-29

**Authors:** Jamie W Sinton, Amol Pednekar

**Affiliations:** 1 Anesthesiology, Cincinnati Children’s Hospital Medical Center, Cincinnati, USA; 2 Radiology, Cincinnati Children’s Hospital Medical Center, Cincinnati, USA

**Keywords:** pre-ejection period, respiratory sinus arrhythmia, heart defects, autonomic nervous system, pediatric congenital heart disease, congenital heart disease, cardiac magnetic resonance (cmr)

## Abstract

Perioperative autonomic nervous system (ANS) measurements are evolving toward increasing import and utility. We present a three-year-old male with Down Syndrome who underwent ambulatory autonomic monitoring during surgery followed by cardiac magnetic resonance (CMR) imaging. Autonomic data from both environments are compared to age-related norms. We are the first to describe a method for acquiring and trending autonomic data from clinically indicated CMR scans in order to monitor autonomic function. These data are proof of concept for the use of routinely collected CMR data as a surrogate for autonomic data in children, noting differences in the autonomic effects of anesthetic techniques.

## Introduction

The autonomic nervous system (ANS) is a complex control system that maintains a homeostatic state by automatically regulating many bodily processes, including heart and respiratory rate. Physiologic exposure to anesthetic agents has recognizable ANS effects. Therefore, the monitoring and management of the ANS during anesthesia are paramount.

The utility of autonomic function measurement is growing. Historically, autonomic function measurement has often been unavailable. When available, it was often too invasive for routine monitoring. However, technological and research advances have increased the availability and utility of autonomic function measurement [1,2]. Age-related normative values for ANS measurements during maturation have become available [3].

Respiratory sinus arrhythmia (RSA) is a commonly used measure of parasympathetic nervous system activity. RSA is the heart rate increase and decrease that occur during the respiratory cycle. Sympathetic nervous system activity can be measured using the pre-ejection period (PEP), which represents the sympathetically mediated time from the onset of ventricular depolarization to the ejection of blood into the aorta. Autonomic measurements of PEP and RSA can be obtained or estimated from both ambulatory autonomic monitoring and cardiac magnetic resonance (CMR) imaging.

This presented case is the first report to describe a method for acquiring and trending autonomic data from clinically indicated CMR scans in order to monitor autonomic function. We obtained RSA and an estimate of PEP in a child under general anesthesia during a clinically indicated CMR scan and compared it with recent ambulatory ANS data from the Vrije Universiteit Ambulatory Monitoring System (VU-AMS) (Amsterdam, Netherlands {http://www.vu-ams.nl/, not approved for clinical use by the Food and Drug Administration}). Autonomic function was measured perioperatively using VU-AMS. Similar data were extracted from his clinically indicated CMR imaging. Data from elective perioperative autonomic monitoring were compared to data routinely obtained, although rarely analyzed, from a CMR scan.

## Case presentation

A three-year-old, 13.5-kilogram, American Society of Anesthesiologists physical status 3 male presented for outpatient adenotonsillectomy and surveillance echocardiography. His history includes trisomy 21 (Down Syndrome) and complete atrioventricular canal defect status post repair. His preoperative RSA was 37.5 milliseconds. During his initial anesthetic for adenotonsillectomy and surveillance echocardiogram, he was maintained on sevoflurane, and his RSA was 19.75 milliseconds. Unexpectedly, the child’s echocardiogram was significant for a noted fluid collection compressing the right ventricular outflow tract. He was admitted to the hospital, and six days later, he underwent CMR (Ingenia 1.5T, Philips Healthcare, Best, Netherlands) to characterize this fluid collection. During propofol anesthesia for the CMR scan, his RSA was 44 milliseconds (with the caveat that he was in the CMR environment) [4]. Anatomic, functional, angiographic, and quantitative flow images were acquired using vectorcardiography (VCG) and respiratory bellows for physiologic synchronization as in a routine cardiac imaging session.

We acquired anatomic, functional, angiographic, and quantitative flow magnetic resonance (MR) images. Cine images (frame rate of one image per 29 milliseconds) were produced to assess ventricular function and aortic flow. Portions of the three-dimensional anatomic CMR imaging data were acquired over 120-millisecond intervals during the quiescent diastolic cardiac phase, determined by VCG signal and exhalation by the bellow signal. These signals were obtained at the sampling rate of 500 hertz and were accessed retrospectively via an institutional research agreement between the hospital and Philips Healthcare. The signals were analyzed to compute R-R intervals over 390 seconds during the three-dimensional anatomic image acquisition.

During the CMR scan, RSA was obtained from respiratory bellow signals. RSA was calculated by the difference between average R-R intervals on the VCG between inspiration and expiration. PEP was computed from an electrocardiogram (ECG) on the day of initial anesthesia (during which his heart rate was 82 beats per minute). The Q-R interval was found to be 48 milliseconds. The PEP was found to be 92 milliseconds. Autonomic data obtained from the VU-AMS and CMR were compared to normal values (see Table 1) [3,5].

**Table 1:** Autonomic Nervous System Data (VU-AMS, CMR Data, and Normal Values) VU-AMS: Vrije Universiteit Ambulatory Monitoring System; CMR: cardiac magnetic resonance; RSA: respiratory sinus arrhythmia; msec: milliseconds; PEP: pre-ejection period; ml: milliliters; LV: left ventricular ---Not applicable *Obtained from most recent electrocardiogram, 44 msec is Q wave onset to R peak from most recent electrocardiogram, and 48 msec is the duration of time from the R peak to the opening of the aortic valve on CMR **Bullet, method for measuring ventricular volume by echocardiogram ***Stroke volume calculated based on the formula provided by Buechel and colleagues [5] ****Duration of aortic valve opening

	VU-AMS	CMR	Normal Values [3]
Breathing control	Assisted via facemask	Nasal canula	Spontaneous
RSA (msec)			
Baseline	37.5	---	43
Induction	19.75	44	---
PEP (msec)			
Baseline	96.7	---	66
Induction	91	44 + 48*	---
Stroke volume (ml)			
Baseline	18.2	---	19**
Induction	19.2	22	22.4***
Tidal volume (ml)			
Baseline	98.3	---	---
Induction	133.5	---	---
LV ejection time (msec)			
Baseline	286.4	---	---
Induction	288.6	300****	---

Prior to his general anesthesia, his mother consented to his participation in a prospective study involving the collection of VU-AMS data and later provided written Health Insurance Portability and Accountability Act of 1996 (HIPAA) authorization allowing this data to be submitted for publication. A comparison of that study data with acquired data from clinically indicated CMR imaging was used with CAse REport (CARE) guidelines [6] to construct this case report.

## Discussion

The VU-AMS was developed at the Vrije Universiteit in Amsterdam and is used worldwide by research groups in a variety of settings. Over 100 publications feature the VU-AMS in research on a range of topics and in samples that include young children, adolescents, and adults. The VU-AMS is novel to ANS monitoring; its measurements represent an enormous paradigm shift from episodic single ANS function data, such as urine catecholamines, to large data sets of continuous, non-invasive characterization of the autonomic physiology. The VU-AMS allows for recording autonomic and cardiovascular activity in various research settings, including ambulatory monitoring in naturalistic settings. It has been tested in young children, adolescents, and adults. Due to its non-invasive character, the VU-AMS can be used in almost all subjects, including vulnerable groups such as very young children (age <1 year) and pregnant females and issues with intellectual disabilities. It operates on two AA batteries and allows 24-48 hours of recording time.

**Figure 1:**
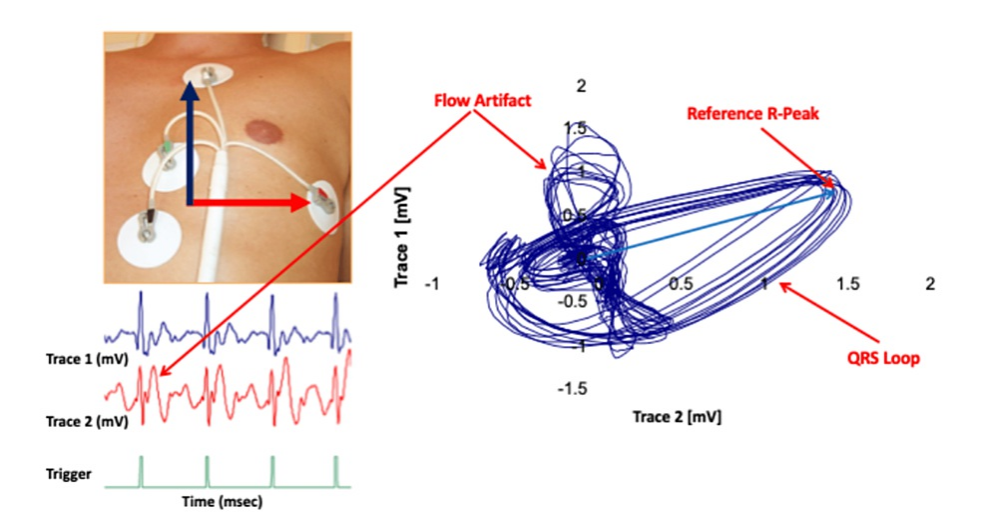
Vector Cardiogram Tracing The velocity of blood exiting the semilunar valves during ventricular systole creates a magneto-hemodynamic effect as blood courses through the ascending, transverse, and descending aortic arch

During every CMR, vectorcardiography (VCG) is used for cardiac synchronization [7]. A bellow is placed over the child’s xiphoid process for respiratory synchronization. The VCG, like ECG, is derived from cardiac electrical activity. VCG is used instead of ECG out of necessity. The electrical voltage signal of the heart is corrupted by the voltage generated due to magneto-hemodynamic effects of accelerating blood exiting the semilunar valves at each ventricular systole. In VCG, electrical voltages are measured along two orthogonal axes and combined as a vector to form the ovular VCG signal trace, as shown in Figure 1. This cardiac electrical activity monitoring method allows for robust detection of R waves.

Comparisons of parasympathetic tone via RSA were consistent with the known effects of general anesthesia and CMR. During his initial anesthetic, the patient was maintained on sevoflurane. His RSA was 37.5 milliseconds while conscious and 19.75 milliseconds during anesthesia induction (during which he was anxious but cooperative). This is consistent with sevoflurane’s known parasympatholytic effect [8].

During his MRI, the patient was maintained on a propofol infusion. The child’s RSA lengthened to 44 milliseconds during general anesthesia with propofol and in the MRI environment. The preservation of RSA during propofol infusion may coexist with propofol’s known depressant effects on sympathetic nervous tone, although its direct impact on parasympathetic tone is unavailable [9].

**Figure 2:**
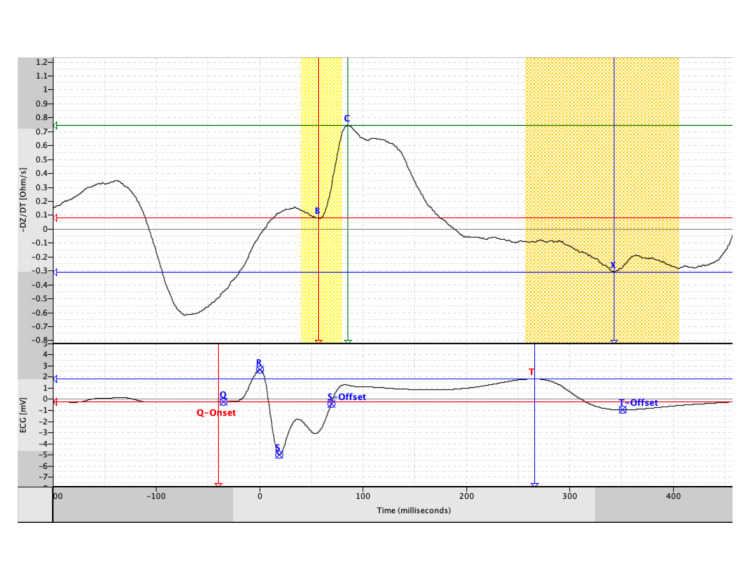
Impedance Cardiogram Image Produced by VU-AMS for the Calculation of Pre-ejection Period The pre-ejection period is computed as the time from the onset of the Q wave on the electrocardiogram (ECG) (see bottom trace) to the time that the aortic valve opens (see top trace at point B). Impedance cardiogram is graphed as the negative change in (first derivative) thoracic bioimpedance versus time

PEP measures sympathetic function, the time from onset of depolarization to aortic valve opening. It is measured by the VU-AMS and can be estimated from cine magnetic resonance (MR) images of the aortic root. The VU-AMS device measures the time interval from the Q wave onset on the ECG to the B point (opening of the aortic valve) of the impedance cardiogram. See Figure 2 for graphical depiction.

PEP is estimated from the cine quantitative flow of MR images from the aortic root over time from the recorded R peak wave. This interval is not well characterized in children but is thought to be relatively constant over a range of heart rates. The time of the opening of the aortic valve with respect to R peak wave was available. However, the time of the Q wave onset cannot be determined by VCG in an MR environment due to the magneto-hemodynamic effect. This time from the R peak wave to the aortic valve opening lacks the time from the onset of the Q wave to the R peak wave to complete the PEP. Therefore, direct determination of PEP with VCG during MRI is impossible.

However, PEP can be estimated by using a prior ECG. This patient’s PEP (92 milliseconds) was computed from an ECG on the day of his initial anesthetic, during which his heart rate was 82 beats per minute. This Q-R interval was found to be 48 milliseconds. Figure 2 shows an image of impedance cardiogram VU-AMS data for the calculation of the PEP. The PEP is computed as the time from the onset of the Q wave on ECG (see bottom trace) to the time that the aortic valve opens (see top trace at point B).

This patient had stroke volume calculations by VU-AMS, CMR, and an echocardiogram. Nederend and colleagues recently updated a stroke volume calculation [10]. Stroke volume is computed in CMR using cine images of the ventricle and as an area under the curve of aortic flow versus the time from quantitative flow CMR images. Tidal volume is not directly measured by routine CMR and depends on anesthetic and airway obstruction, as well as the child’s age. The anesthetic record for the child’s MRI did not disclose a tidal volume. Tidal volume by VU-AMS is shown in Table 1.

Both VU-AMS and CMR quantify left ventricular ejection time (LVET). VU-AMS measures the duration of the reduction in thoracic bioimpedance as blood is ejected into the great vessels. The VU-AMS measured this patient’s LVET as 308 milliseconds. On MRI, LVET is measured as the duration of flow out of the aortic valve. CMR measured this patient’s LVET as 300 milliseconds. One possible reason for the discrepancy between these two values is the frame rate of the quantitative flow MR images (29 milliseconds per image). Additionally, quantitative flow MR images are not instantaneous. They are generated by combining portions of imaging data acquired over 60 heartbeats, representing the average flow pattern over 60 cardiac cycles.

Adults with Down Syndrome are known to have sympathetic failure, which would be manifested as a lengthening of PEP compared to typically developed adults. At what age this occurs and to what degree have yet to be characterized [2]. For these reasons, precision medicine, using each patient as his own control and trending autonomic values over time, may document heart disease trajectories (albeit with impact due to factors other than heart disease).

The strength of this case report is that meaningful, autonomic data beyond RSA may be obtainable during routine clinical care with CMR. The main limitation of using VCG data acquired within the MR environment to calculate PEP is that it cannot be precisely calculated because only the duration from the R peak wave to the opening of the aortic valve is available (rather than from the onset of the QRS complex). The main weakness of our comparison was that VU-AMS and CMR data were not obtained simultaneously, making direct comparisons impossible.

## Conclusions

As previously stated, changes in autonomic function predict adverse cardiac events in adults. Additionally, corrective cardiac procedures result in measurable autonomic changes in children as do routine growth and maturation. Consequently, the concept of ANS monitoring through routinely collected data is relevant. Trending of autonomic function parameters via clinically indicated CMR scans over time may be possible. More work is needed to validate this idea.
